# The influence of common polygenic risk and gene sets on social skills group training response in autism spectrum disorder

**DOI:** 10.1038/s41525-020-00152-x

**Published:** 2020-10-12

**Authors:** Danyang Li, Nora Choque-Olsson, Hong Jiao, Nina Norgren, Ulf Jonsson, Sven Bölte, Kristiina Tammimies

**Affiliations:** 1 Center of Neurodevelopmental Disorders (KIND), Centre for Psychiatry Research, Department of Women’s and Children’s Health, Karolinska Institutet, Stockholm County Council, Stockholm, Sweden; 2 Astrid Lindgren Children’s Hospital, Karolinska University Hospital, Region Stockholm, Stockholm, Sweden; 3 Child and Adolescent Psychiatry, Stockholm Health Care Services, Region Stockholm, Stockholm, Sweden; 4 Center for Psychiatry Research, Department of Clinical Neuroscience, Karolinska Institutet and Stockholm Health Care Services, Region Stockholm, Stockholm, Sweden; 5 Department of Biosciences and Nutrition, Karolinska Institutet, and Clinical Research Centre, Karolinska University Hospital, Huddinge, Sweden; 6 Department of Molecular Biology, National Bioinformatics Infrastructure Sweden (NBIS), Science for Life Laboratory, Umeå University, 901 87 Umeå, Sweden; 7 Department of Neuroscience, Child and Adolescent Psychiatry, Uppsala University, Uppsala, Sweden; 8 Curtin Autism Research Group, School of Occupational Therapy, Social Work and Speech Pathology, Curtin University, Perth, WA Australia

**Keywords:** Autism spectrum disorders, Molecular medicine

## Abstract

Social skills group training (SSGT) is a frequently used behavioral intervention in autism spectrum disorder (ASD), but the effects are moderate and heterogeneous. Here, we analyzed the effect of polygenic risk score (PRS) and common variants in gene sets on the intervention outcome. Participants from the largest randomized clinical trial of SSGT in ASD to date were selected (*N* = 188, 99 from SSGT, 89 from standard care) to calculate association between the outcomes in the SSGT trial and PRSs for ASD, attention-deficit hyperactivity disorder (ADHD), and educational attainment. In addition, specific gene sets were selected to evaluate their role on intervention outcomes. Among all participants in the trial, higher PRS for ADHD was associated with significant improvement in the outcome measure, the parental-rated Social Responsiveness Scale. The significant association was due to better outcomes in the standard care group for individuals with higher PRS for ADHD (post-intervention: *β* = −4.747, *P* = 0.0129; follow-up: *β* = −5.309, *P* = 0.0083). However, when contrasting the SSGT and standard care group, an inferior outcome in the SSGT group was associated with higher ADHD PRS at follow-up (*β* = 6.67, *P* = 0.016). Five gene sets within the synaptic category showed a nominal association with reduced response to interventions. We provide preliminary evidence that genetic liability calculated from common variants could influence the intervention outcomes. In the future, larger cohorts should be used to investigate how genetic contribution affects individual response to ASD interventions.

## Introduction

The response to behavioral interventions varies between individuals with the same neurodevelopmental and psychiatric disorders. One of the factors contributing to this heterogeneity might be genetic predisposition^1^. Genome-wide association studies (GWAS) have been used to pinpoint common variants, with mostly small effects, associated with disorders or personality traits across the genome. For behavioral interventions, the size of available cohorts with detailed outcomes is limited, and to date, no significant variants have been associated with any specific intervention outcome using GWAS^2^. However, the use of polygenic risk score (PRS), an aggregate measure of the cumulative effects of single nucleotide polymorphisms (SNPs) derived from GWAS, has provided promising results in psychiatry both for behavioral and pharmacological treatments^3–7^. For instance, PRS has been studied to predict the response to cognitive behavior therapy in major depressive disorder (MDD)^4^. In addition to PRS, gene-set analysis can be utilized to group multiple genetic variants in genes and further to related gene sets to unravel biological processes and cellular functions related to intervention responses^8^. Relevant gene-set associations have been identified for interventions in psychiatry^8–12^. For example, genetic variations in genes underlying glutamatergic or NMDA neurotransmission have been implicated in short-term antipsychotic medication efficacy in schizophrenia, and the calcium signaling pathway has been indicated to respond to selective serotonin reuptake inhibitors in obsessive-compulsive disorder^10,12^.

Autism spectrum disorder (ASD) is a neurodevelopmental disorder characterized by impairments in social communication and interaction, together with restricted, repetitive behaviors^13^. ASD commonly co-exists with other neurodevelopmental and psychiatric disorders such as attention-deficit hyperactivity disorder (ADHD), anxiety, depression, and intellectual disability^14^. Genetic knowledge of ASD has increased rapidly in recent years. For a number of autistic individuals, rare genetic variants, such as loss-of-function variants and copy number variations (CNVs) in specific genes and loci, can indicate a molecular etiology^15^. In addition, common variants have been shown to play a role in the disorder^16–18^. Although there are only a few genome-wide significant SNPs identified for ASD, cumulative polygenic variation summarized by PRS has shown to be predictive of ASD and autistic traits in the general population^18,19^. Studies have also indicated shared genetic liability of polygenic risk in ASD and psychiatric disorders^20,21^. Educational attainment (EA), defined as the highest degree of education, also has a confirmed genetic correlation with ASD^22^, and academic achievement has been linked to social skills^23^. Both rare and common genetic variations in ASD have shown to converge on specific gene sets such as synaptic formation and targets of Fragile-X mental retardation protein (FMRP)^24,25^. To date, no studies have investigated how the already implicated common genetic variation in ASD or related diagnoses and traits would relate to intervention outcomes in autistic individuals.

Social skills group training (SSGT) is one of the most frequently used behavioral interventions in ASD, aiming to alleviate social communication difficulties in verbal individuals within the normative intellectual range in a group setting^26^. The largest randomized controlled trial (RCT) of SSGT (KONTAKT) to date, conducted by our center in Sweden, included children and adolescents with ASD and at least one common neurodevelopmental or psychiatric comorbidity^26^, such as ADHD, anxiety, and depression. In the trial, SSGT as an add-on to standard care was found to have a small to moderate effect compared to standard care only, with significant effects on the primary outcome limited to adolescents (13–17 years) and females^26^.

We recently showed that autistic individuals who were carriers of clinically significant and rare genic CNVs larger than 500 kb had significantly inferior outcomes after SSGT within the RCT^27^. Here, we expand our genetic investigations to test the association between SSGT and standard care intervention responses in ASD and common variants using PRS and gene-set analysis. We hypothesized that PRSs for ASD, ADHD, and EA, as well as common variants within known ASD gene sets, would influence intervention outcomes. Additionally, we tested if there was a significant correlation between PRSs with clinically significant CNVs and the clinical measures of parent-reported Social Responsiveness Scale (SRS), ADHD diagnosis, and IQ in our ASD cohort. We also tested if different PRSs were correlated with each other in our cohort. To the best of our knowledge, there are no studies published that have evaluated the influence of common variants on intervention response in ASD or neurodevelopmental disorders. Our results will provide further evidence of the potential use of genetic profiles to predict individual-level outcomes for ASD interventions.

## Results

### Sample characteristics

Autistic children and adolescents from the multicenter, randomized pragmatic RCT of SSGT (KONTAKT) in Sweden, were included in this study^26^. Each participant received standard care intervention and was randomized to the active SSGT group, to additionally receive SSGT, or to the control group. Saliva samples from 207 participants were collected for genotyping during the RCT^27^. After quality control (QC) for this study, 188 participants were analyzed for common variants. Of these, 169 participants had outcome data at post-intervention, and 152 participants at follow-up, and 99 participants belonged to the active SSGT group. When comparing individuals of this RCT subgroup with the total clinical cohort^26^, we did not detect any differences in characteristics of sex, age, and IQ (Supplementary Table 1). The final genotype data consist of 539,106 SNPs after genotyping marker QC and 5,126,694 SNPs after imputation QC.

### Correlation between PRSs and related characteristics

We chose three phenotypes: ASD, ADHD, and EA to calculate PRSs separately using five *P* value thresholds (Pts) (<0.01, <0.05, <0.1, <0.5, and <1) from GWAS summary results^18,28,29^. The correlation between ADHD comorbidity status and PRS for ADHD, pre-intervention parent-reported SRS, clinically significant CNVs of ASD and PRS for ASD, as well as IQ and PRS for EA, was tested (Supplementary Fig. 1). No correlation between ASD PRS and baseline SRS or carrier status of clinically significant CNVs was observed (Supplementary Fig. 1a, b). Autistic participants with ADHD comorbidity had higher ADHD PRS compared with participants without ADHD (Pt_0.1_, *P* = 0.010; Pt_0.5_, *P* = 0.0075; Pt_1.0_, *P* = 0.0077) (Supplementary Fig. 1c), and IQ was positively correlated with PRS for EA (Pt_0.05_, *P* = 0.038; Pt_0.1_, *P* = 0.024; Pt_0.5_, *P* = 0.014; Pt_1.0_, *P* = 0.012) (Supplementary Fig. 1d). Correlations of PRSs for ASD, ADHD, and EA were performed using selected Pt of each PRS (Supplementary Fig. 2). PRS for ADHD was positively correlated with PRS for ASD (*r* = 0.41, *P* = 1.7 × 10^−10^) but was negatively correlated with PRS for EA (*r* = −0.22, *P* = 0.00099). No correlation was found between ASD PRS and EA PRS (*r* = 0.039, *P* = 0.56).

### Proportion of variance in intervention outcomes explained by PRS

**Fig. 1:**
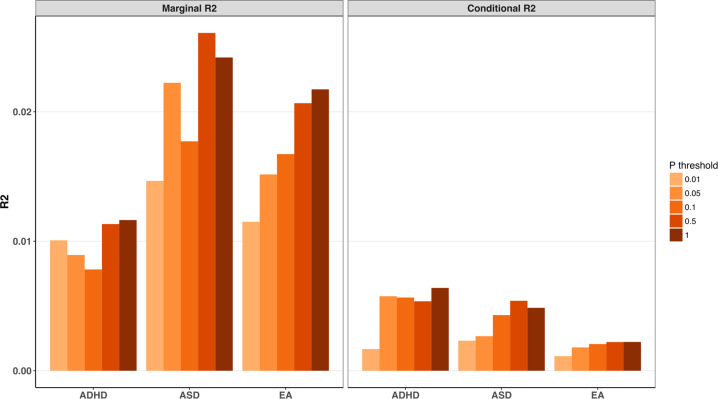
Proportion of variance explained (R^2^) by polygenic risk scores (PRSs) of autism spectrum disorder (ASD), attention-deficit hyperactivity disorder (ADHD), and education attainment (EA) in intervention outcomes. PRS were derived using five *P* value thresholds (<0.01, <0.05, <0.1, <0.5, <1) and the presented results are from the model adjusted for clinically significant rare copy number variations (CNVs). Marginal R^2^ and conditional R^2^ were calculated representing the variance explained by only fixed effects as well as the sum of fixed and random effects.

To investigate the association of intervention outcomes, measured by the parent-reported SRS^30^ at pre-intervention, post-intervention, and follow-up, with the three different phenotype PRSs, we used mixed linear model (MLM) with interaction effect of PRS × time × intervention adjusting for additional fixed factors such as age, sex, the most significant four principal components of participants ancestry, and random factors including clinical centers and each individual^26^. Additionally, we adjusted for carrier status of clinically significant CNVs and rare large size CNVs (>500 kb)^27^ separately in statistical models. First, we calculated marginal R^2^ and conditional R^2^ to explain the role of PRS on the variance of fixed and total effects of treatment outcome, respectively^31^. In the model examining the impact of PRS for ASD, the largest explained marginal and conditional variance in the outcome was Pt_0.5_ when controlling for clinically significant CNVs (Fig. 1). We detected small differences in the explained variance when testing for ADHD PRS at all Pts, with the highest value at Pt_1.0_ (Fig. 1). In EA PRS, increasing values of marginal and conditional R^2^ occurred with higher Pt, in which Pt_1.0_ explained the most variance. All three tested models controlling the carrier status of CNVs showed similar results (Fig. 1 and Supplementary Fig. 3).

### Association between PRS and intervention outcomes

**Fig. 2:**
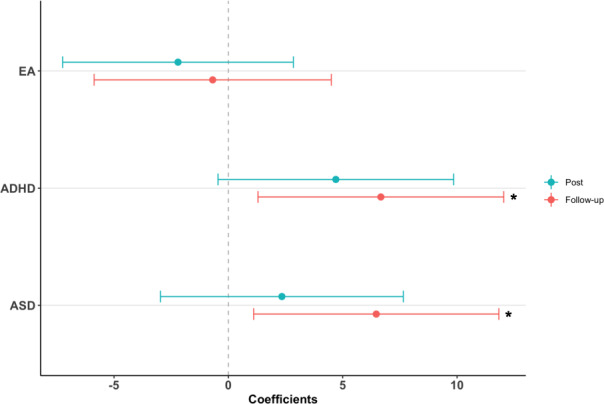
Association of polygenic risk score (PRS) for autism spectrum disorder (ASD), attention-deficit hyperactivity disorder (ADHD), and educational attainment (EA) in intervention outcomes at post-intervention and follow-up. Correlation coefficients are shown with 95% confidence intervals. Different *P* value thresholds which have highest explained variance for each PRS were included in the model (ASD: Pt = 0.5, ADHD: Pt = 1, EA: Pt = 1). Clinically significant rare copy number variations (CNVs) were added as a cofactor in the model. *P < 0.05.

We used the PRS Pt with the highest explained variance for each phenotype (ASD, ADHD, and EA) in our analyses to investigate the association with the intervention outcomes (Fig. 2). When contrasting the intervention outcomes and the PRS effect using three-way interaction MLM (PRS × time × intervention), we showed inferior outcome after SSGT for individuals with higher PRS for ASD at follow-up (*β* = 6.47, *P* = 0.019). However, the association was not significant after multiple testing correction. Similarly, participants with higher ADHD PRS improved less after SSGT in comparison with the participants in the standard care group at follow-up (*β* = 6.67, *P* = 0.016), and the significance remained after multiple testing correction. When analyzing only the effect of PRS using two-way interaction of PRS × time, higher PRS for ADHD was associated with a decrease in parental-rated SRS, indicating overall better outcomes (post-intervention: *β* = −4.747, *P* = 0.0129; follow-up: *β* = −5.309, *P* = 0.0083, Supplementary Table 2). The associations remained significant after adjusting for ADHD comorbidity (Supplementary Table 2). No significant associations were found for EA PRS (Fig. 2). No difference was found when we compared the model adjusting for large size rare CNVs nor the model without CNV adjustment (Supplementary Table 2). Secondary results of all Pts for ADHD and ASD PRSs in three models were shown in Supplementary Table 2.

To investigate further the differential effects between the intervention groups, we computed the MLMs separately in the SSGT and standard care groups using two-way interaction PRS × time, and demonstrated that individuals with higher PRS for ASD showed less improvement in the SSGT group at follow-up as indicated in the main model (post-intervention: *β* = 0.430, *P* = 0.835; follow-up: *β* = 5.145, *P* = 0.0144). In contrast, no significant effect for PRS ADHD was found in the SSGT group, but higher ADHD PRS was associated with significant improvement effect in the standard care group (post-intervention: *β* = −4.729, *P* = 0.00647; follow-up: *β* = −5.277, *P* = 0.00394, Supplementary Table 3).

To identify if any interaction between PRS and clinically significant CNVs, secondary linear model with interaction effect PRS × clinically significant CNVs, showed changes in the parent-reported SRS when accounting for ASD PRS was similar in noncarriers and carriers of clinically significant CNVs, while PRS for ADHD showed a modest interaction with clinically significant CNVs on the SRS changes without statistical significance (Supplementary Fig. 4).

### Gene sets association with intervention outcomes

In addition to PRS, we tested if common genetic variation in selected gene sets could explain the intervention outcomes using competitive gene-set analysis. Thirty-two gene sets within five categories (synaptic, glial, FMRP, glutamate, and mitochondrial) were included from other studies^25,32–37^. A linear regression model was built using changes of parent-reported SRS score between post-intervention or follow-up and pre-intervention as outcomes, adding age, sex, intervention groups, CNV carrier status (large size CNVs and clinically significant CNVs), and four largest principal components as cofactors. None of the gene sets results were significant after multiple testing correction. Nominally significant effects were found in four gene sets causing inferior outcomes at posttreatment: intracellular signal transduction (large size CNVs: *β* = 0.204, *P* = 0.0027; clinically significant CNVs: *β* = 0.202, *P* = 0.0029), cell adhesion and trans-synaptic signaling (large size CNVs: *β* = 0.255, *P* = 0.0071; clinically significant CNVs: *β* = 0.247, *P* = 0.0090), excitability (large size CNVs: *β* = 0.268, *P* = 0.017; clinically significant CNVs: *β* = 0.247, *P* = 0.026), GPCR signaling (large size CNVs: *β* = 0.255, P = 0.023; clinically significant CNVs: *β* = 0.252, *P* = 0.024), all belonging to the synaptic group (Table 1). For follow-up, only RNA and protein synthesis, folding and breakdown (large size CNVs: *β* = 0.184, *P* = 0.030; clinically significant CNVs: *β* = 0.185, *P* = 0.030) in synaptic group showed similar effects (Table 1). The results for other gene sets did not reach nominal significance (Supplementary Table 4).

**Table 1:** Effect of most significant gene sets (*P* < 0.05) on intervention outcomes at post-intervention and follow-up adjusted for clinically significant rare copy number variations (CNVs) and large size (>500 kb) rare CNVs.

Gene sets	Gene-set groups	Number of genes	Clinically significant rare CNVs				Large size rare CNVs			
			Beta	Se	*P*	Corrected *P*	Beta	Se	*P*	Corrected *P*
Post-intervention										
Cell adhesion and trans-synaptic signaling	Synaptic	74	0.247	0.104	0.0090	0.2340	0.255	0.104	0.0071	0.1988
Excitability	Synaptic	55	0.247	0.127	0.0262	0.5383	0.268	0.127	0.0174	0.4110
GPCR signaling	Synaptic	40	0.252	0.128	0.0244	0.5142	0.255	0.128	0.0231	0.5011
Intracellular signal transduction	Synaptic	138	0.202	0.073	0.0029	0.0837	0.204	0.073	0.0027	0.0832
Follow-up										
RPSFB	Synaptic	62	0.185	0.098	0.0303	0.5871	0.184	0.098	0.0305	0.5928

*GPCR* G-protein-coupled receptor, *RPSFB* RNA and protein synthesis, folding and breakdown.

## Discussion

In this study, by calculating PRS and analyzing related gene sets, we tested if different subsets of common variants and biological gene groups would influence the outcome of SSGT and standard care interventions. Combining the MLM results from all participants in the SSGT trial KONTAKT and analyzing the outcomes of the different intervention subgroups, we showed that a higher common genetic variant load for ADHD and ASD was negatively associated with SSGT response in comparison with standard care in autistic individuals. Interestingly, the differential effect of PRS for ADHD within the intervention groups was due to significant improvement in autistic individuals with higher PRS for ADHD in the standard care group. The effects of PRS models remained even when we controlled for rare CNV carrier status of individuals suggesting an independent role of PRS on intervention outcome. Overall, the identified effects of PRS were modest compared to our earlier results on the effect of rare clinically significant and large CNVs^27^. We also demonstrated significant correlations between PRS for ADHD and ADHD comorbidity and PRS for EA and IQ levels in our study cohort. In the three tested phenotypes, we found that there was a positive correlation between PRS for ADHD and ASD and a negative correlation between PRS for ADHD and EA. Additionally, we showed suggestive evidence that genes involved in synaptic functions could be important for modulating response to ASD interventions, as several gene sets important for synaptic functions showed nominal significance to influence outcome.

Given the high heterogeneity of intervention responses among individuals with ASD, it is important to find factors influencing the outcome, which could be used to tailor interventions for each individual. Our results provided intriguing evidence, although very preliminary, that in addition to the rare high impact CNVs, polygenic contribution of common variants could have a moderating role on intervention outcomes in autistic individuals, especially ADHD PRS. Although our main analyses contrasting the outcomes after SSGT with outcomes in standard care group showed less improvement for individuals with higher PRS for ADHD receiving SSGT, the subgroup analyses revealed that this difference was due to greater improvement after standard care for individuals with higher PRS for ADHD. Interestingly, the effect of PRS for ADHD remained when adjusting for clinical ADHD comorbidity. Shared genetic liability between ASD and ADHD has been reported in family studies^38,39^, and genetic overlap between the two neurodevelopmental conditions is evident from genome-wide correlation analysis showing similar tendency with PRS correlation as found in our result^22,40,41^. PRS for ADHD has also been used to predict ASD-related measures such as pragmatic language abilities^42^. Compared to ASD alone, having ASD and ADHD symptoms was confirmed to be associated with greater impairments in socialization adaptive skills in clinical presentation^43^. Based on genetic sharing between the two conditions, our results highlight the connection between ADHD genetic information and ASD intervention effects. Furthermore, the correlation between PRS and ADHD comorbidity indicates that ADHD PRS could be considered to evaluate liability to ADHD in individuals first diagnosed with ASD.

Compared to the results of PRS for ADHD, we found an only indicative association between PRS for ASD and intervention outcomes. Currently, it is not known if the same genetic factors would influence the risk of developing ASD and intervention response measured here. Unlike Grove et al.^18^ who reported that ASD PRS with Pt_0.1_ could predict ASD diagnosis, the largest explained variance in our study was at Pt_0.5_, which putatively suggests that additional genetic variants from a less stringent Pt play a role in how autistic individuals respond to interventions. In other disorders such as MDD, studies have found variants associated with MDD can be either positive or negative for the outcome of antidepressants^44^, and the results of PRS as a predictor of treatment outcome are inconsistent^45,46^. Further studies should clarify how ASD PRS might modulate intervention outcomes. Variants which confer risk to ASD should also be tested for their role in interventions if larger cohorts are available.

We did not find any association between PRS for EA and intervention responses, and our power calculation showed that a significantly larger sample size would be needed to estimate the PRS EA effects robustly. Moreover, we did not detect any correlation between PRS for EA and ASD in our sample. Studies using genetic correlation analysis corroborated interrelated results between ASD and years of education and college attainment^22,47^. Some PRS studies suggest that PRS for ASD is associated with EA^47^, and EA PRS is associated with lower externalizing behaviors^48^. Still, no study has shown the prediction of autistic traits or ASD-related interventions using PRS for EA. As expected, we found a positive correlation between PRS EA and IQ in our sample, which is in accordance with other findings in the general population^49^.

Interestingly, the inferior outcome of SSGT compared with standard care for individuals with higher PRSs for ASD and ADHD seemed to be particularly pronounced at follow-up. A similar tendency was previously also seen in individuals with clinically significant rare CNVs^27^. This suggests that additional care during or after SSGT treatment should be considered for individuals with higher genetic risk to alleviate the genetic influence on intervention outcome. Recently, a long version of SSGT intervention (24 weeks) was conducted, resulting in larger positive effects compared to 12-week intervention^50^. Further studies should investigate if genetic effects remain when the intervention dose is increased.

Although no robust association after multiple testing corrections were shown for the gene-set analysis, we found suggestive evidence that genes necessary for synapse formation and maintenance may influence intervention responses. Since synapse formation and synaptic plasticity have been indicated as one of the key neuronal mechanisms in ASD, differences in these key steps in brain development could play a major role in how individuals respond to interventions^51^. Some aspects of treatment-induced behavior improvement are also related to brain plasticity changes in psychiatric disorders. For instance, significant increases of gray matter in the left hippocampus and left amygdala correlated with the degree of improved cognition are found in early onset schizophrenia individuals after 2 years of social skills group therapy and cognitive remediation^52^.

We acknowledge the limitations of our study. First, the sample size from RCT is limited, which restricted the precision of our analyses and precluded in-depth analysis of complex pathways and single variants. Indeed, this is a preliminary study on detecting the role of common variants on ASD intervention. Nevertheless, results in our study can pinpoint an exploratory direction to inspire more genetic research to focus on heterozygous intervention effects of ASD and neurodevelopmental disorders. Furthermore, we provide power prediction on different sample sizes to help studies make future plans to identify the PRS effect on interventions in ASD. Second, the analyzed sample included individuals with normative IQ and common neurodevelopmental and psychiatric comorbidities, limiting the generalizability across the total population of autistic individuals. Finally, studies have shown that loci harboring common alleles are also enriched for rare variants with large effects from whole-exome sequencing in psychiatric disorders^53^. Although we have adjusted for the carrier status of rare CNVs, other rare variants were not controlled for in this study. In future, more genetic information can be combined to better understand their effect on intervention outcomes.

In conclusion, this is the first study showing that common polygenic risk, especially for ADHD, could play a role in intervention outcomes for autistic individuals, and that gene sets from synaptic groups may play a potential biological role in the response. Replications, including larger sample sizes and a combination of more genetic and clinical factors, are needed to further clarify the genetic influences and mechanisms behind individual intervention responses.

## Methods

### Study individuals

The original multicenter, randomized pragmatic RCT of SSGT (KONTAKT), recruited participants from 13 child and adolescent psychiatry outpatient units in Sweden between August 2012 and October 2015 (identifier: NCT01854346, registration May 2013). A detailed description of the inclusion and exclusion criteria has been earlier described by Choque Olsson et al.^26^. In short, 296 children (7–12 years) and adolescents (13–17 years) with a diagnosis of autism, atypical autism, Asperger syndrome, or pervasive developmental disorder not otherwise specified using ICD-10 criteria were included in the trial^54^. Based on inclusion criteria for the RCT, all participants had full-scale IQ > 70 according to the Wechsler Intelligence Scale for Children and at least one common comorbid psychiatric diagnosis of ADHD, depression, or anxiety disorder according to ICD-10^54,55^. During the 12-week trial, the standard care group (*N* = 146) received any ongoing support or intervention, and the remaining 150 participants were included in the active SSGT condition. We used the parent-reported SRS as the primary intervention outcome measure, which is a 65-item Likert-type scale generating total scores ranging between 0 and 195, with a higher score indicating greater autism trait severity^30^. SRS was recorded for each individual at baseline (pre-intervention), 12 weeks (post-intervention), and 3 months after the end of the intervention (follow-up).

Participants who contributed saliva samples and had primary outcome measure recorded at either post-intervention or follow-up were included in this study. After selection, clinical data and samples from 207 participants (SSGT group: 105, standard care group: 102) were used for genotyping^27^.

### Ethics

Written informed consent from the parents or legal guardians and verbal consent from the children and adolescents were collected. All the protocols and methods in this study were in accordance with the Declaration of Helsinki. The trial and sample collection were approved by the ethical review board in Stockholm (Dnr 2012/385-31/4) and the clinical authorities of the two involved counties. The trial was registered online (NCT01854346).

### Genotyping and quality control

DNA collection, extraction, and genotyping procedures are described elsewhere in detail^27^. In short, genotyping was done on the Affymetrix CytoScan™ HD microarray platform, containing 743 304 SNP probes, in two separate batches. Data from genotyping were transformed from Affymetrix.CEL format to.tped format using “Affy2sv” package v1.0.14 in R. The position of each SNP was located based on a microarray annotation reference file (version NA32.3). QC of the data using PLINK v1.90 was performed on a per-individual basis within each genotyping batch to remove poorly genotyped individuals and on per-marker QC to exclude low-quality markers following a published protocol^56,57^. Individuals with discordant sex, heterozygosity rate > 3 standard deviation (SD), individual genotype failure rate > 0.03, and relatedness were removed. Ancestry of participants was estimated using principal component analysis based on the HapMap Phase III data using EIGENSOFT v7.2.1^58^. We restricted our analyses to participants with European ancestry. Additionally, the first four principal component values were added in the statistical model to adjust for ancestry. As no batch effects were detected, the qualified data were combined to clean low-quality markers with the following criteria: minor allele frequency < 0.05, Hardy–Weinberg equilibrium < 1 *10-6, individual missingness < 0.1, and marker missingness < 0.05.

### Imputation

We used the 1000 Genomes phase III haplotype data, including individuals from all ancestries, as a reference. SNPs passing QC were separated into autosomes, and haplotypes were inferred based on reference panel using SHAPEIT v2^59^. For each phased autosome, imputation was performed in 5 Mb windows using IMPUTE2 v2.3.2^60,61^. All imputed regions were combined for post-imputation QC. Imputed SNPs were filtered using following metrics: info score < 0.8, minor allele frequency < 0.05, Hardy–Weinberg equilibrium < 1 × 10^−6^, marker missing rate < 0.05, and individual missing rate < 0.1. SNPs were intersected together after post-imputation QC using both SNPTEST v2.5.5 and PLINK v1.90^57,62^.

### Polygenic risk score calculation

To calculate PRSs for ASD, ADHD, and EA, we used the GWAS summary results from Psychiatric Genomics Consortium as reference data (downloaded on July 2018)^18,28,29^. To minimize different population effects, we included only individuals with European ancestry from GWAS reference samples. The estimated odds ratio and *P* value of each SNP allele were used from each reference set. SNPs in both reference and our in-house data were pruned using clumping with a cutoff of *r*^2^ ≥ 0.1 within a 500 kb window. PRS was calculated based on independent SNPs using five Pts (<0.01, <0.05, <0.1, <0.5, and <1) selected from three reference sets, with higher Pt incorporating more SNPs. Allele numbers at each SNP (0, 1, 2), weighted by the natural logarithm of the allelic odds ratio, were summed to calculate an accumulative effect across the genome^3,63^. PRS was then standardized (mean = 0, SD = 1) to test for association. All calculations were conducted using PRSice v2.1.4^64^.

### Gene sets generation

Reference gene sets were obtained based on a previously published study^25^. Thirty-two gene sets within five categories: synaptic (1047 genes), glial (240 genes), FMRP (1809 genes), glutamate (156 genes), and mitochondrial (132 genes) were included^32–37^. SNPs were annotated to genes based on European populations from 1000 genomes and gene locations, Build 37 using MAGMA v1.0.6.

### Statistical analysis

We used an MLM to identify the effect of PRS on SSGT response measured by parent-reported SRS. Three time points (pre-, post-intervention, and follow-up), as well as two study groups (SSGT and standard care), were included to test the interaction effect of PRS × time × intervention as the main reported effect in our analyses. Based on our previous studies^26,27^, factors associated with inferior intervention outcomes were younger age (children), male sex, and CNV carrier status (large size CNVs (>500 kb) and clinically significant CNVs). Therefore, PRS interaction effect, age group, sex, and population stratification of the four largest PCs were added as fixed factors in the model. As to control the influence of ADHD comorbidity in individuals, the comorbidity status of ADHD was included as a fixed factor in the model of PRS for ADHD. Additionally, the clinical centers and each individual were used as random factors. The carrier status of clinically significant CNVs was also added as a fixed factor in the model comparing to the other two models either with large size CNVs or without any CNV adjustment. In MLM, marginal R^2^ considers only the variance of fixed effect, while conditional R^2^ takes both fixed and random effects into account. Therefore, we assessed both marginal R^2^ and conditional R^2^ to explain the role of PRS on the variance of fixed and total effects of treatment outcome, respectively^31^. For each phenotype on each Pt, both marginal and conditional R^2^ were derived from the difference between the model with PRS and the model without PRS, performed by “MuMIn” package v1.43.6 in R^65^. Beta and 95% confidential interval were estimated from MLM to evaluate the interaction effect of PRS with SSGT and standard care at different times. As we tested three PRSs (ASD, ADHD, and EA) in this study, nominal *P* values under 0.0167 were considered significant^66^. MLM was also performed in SSGT and standard care groups separately using two-way interaction PRS × time points including only the main Pts of ASD and ADHD PRS. The relation between SRS changes (post-intervention or follow-up compared to pre-intervention in both SSGT and control groups) and the highest R^2^ PRSs for ASD and ADHD were tested separately for clinically significant CNVs to detect if any difference between carriers and noncarriers. We used the interaction effect of being a carrier of a clinically significant CNV and PRS in linear model using parent-reported SRS changes as the outcome to identify if clinically significant CNVs and PRSs for ASD or ADHD were independent on intervention outcome. In addition, two-sided Student’s *t* test was used to evaluate the difference of PRS for ADHD and ADHD comorbidity status, and PRS for ASD and clinically significant CNVs of ASD. We also performed Pearson correlation to examine the correlation between different PRSs with highest R^2^ in ASD, ADHD, and EA, as well as PRS for EA and IQ level, and PRS for ASD and ASD severity (SRS at pretreatment). All analyses were performed in R v3.4.2.

Bootstrapping was used to evaluate the power of PRS association on intervention outcome. The main PRS of each phenotype were considered for estimation. Samples were randomly selected with replacement based on different specified sizes from our data. We repeated MLM 1000 times to summarize the proportion of significant *P* values (*P* value < 0.05) as power estimation. The power of PRSs for ADHD, ASD, and EA based on our study sample size was 0.766, 0.726, and 0.119, respectively, at a significant level *P* < 0.05. The statistical power of ASD and ADHD PRSs was evaluated to reach 0.90 with sample size > 300 (Supplementary Table 5). All steps were conducted in R v3.4.2.

We used MAGMA v1.0.6 to perform gene and gene-set analysis^67^. Linear regression was chosen to identify the effects of common variation within specific gene sets on intervention outcomes. The changes in the SRS reported by parents between post-intervention or follow-up and pre-intervention were used as regression outcomes. Age, sex, intervention methods, four largest PCs, and CNVs carrier status (large size CNVs and clinically significant CNVs) were added in the model as cofactors. The estimated effect size of competitive test and *P* value on each gene set was obtained, followed by multiple testing correction with positive effect size, indicating a decreased effect of the SSGT response. As no significant gene sets were detected in total samples, we did not perform gene-set analysis in SSGT subgroup.

### Web resources

1000 Genomes Phase III haplotypes reference data: https://mathgen.stats.ox.ac.uk/impute/impute_v2.html#reference.

Psychiatric Genomics Consortium: https://www.med.unc.edu/pgc/.

Gene locations from the NCBI site and SNP locations from 1000 genomes Phase 3 European population Build 37 for MAGMA: https://ctg.cncr.nl/software/magma.

### Reporting summary

Further information on research design is available in the Nature Research Reporting Summary linked to this article.

## Supplementary information

Supplementary Information

Reporting Summary

## Data Availability

The data that support the findings of this study are available on request from the corresponding author and subject to necessary clearances. The raw array and phenotypic data are not publicly available due to limited ethical approval and them containing information that could compromise research participant privacy/consent.

## Code availability

All code in the analyses was based on the original instruction of each software or package and the full set of code is available from the corresponding author upon request. Information about all software and packages was described in the paper.
