# Supplementary material for: The influence of common polygenic risk and gene sets on social skills group training response in autism spectrum disorder
Source: NPJ Genom Med. 2020 Oct 12;5:45. doi: 10.1038/s41525-020-00152-x (PMC7550579; doi:10.1038/s41525-020-00152-x)
Supplement: Supplementary file 1 — Supplementary Information [file 41525_2020_152_MOESM1_ESM.pdf]

The influence of common polygenic risk and gene sets on social skills group training response in autism spectrum disorder

Danyang Li, Nora Choque-Olsson et al.

Supplementary Information

Contents

Supplementary Figures **2**
Supplementary Figure 1 2
Supplementary Figure 2 3
Supplementary Figure 3 4
Supplementary Figure 4 5
Supplementary Tables **6**
Supplementary Table 1. 6
Supplementary Table 2 7
Supplementary Table 3. 11
Supplementary Table 4. 12
Supplementary Table 5. 15

Supplementary Figures

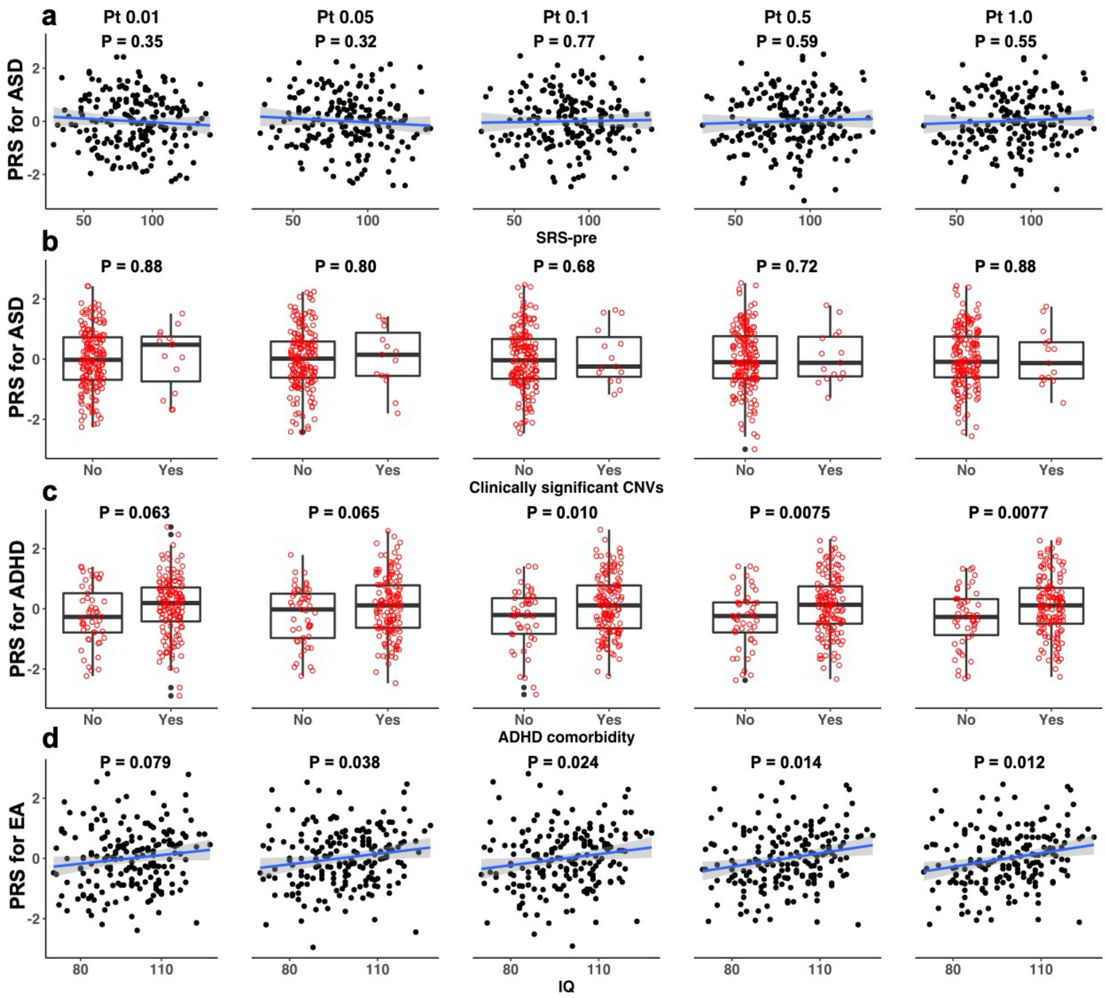

Supplementary Figure 1. Correlation between polygenic risk scores (PRSs) and related characteristics. PRS was calculated on independent SNPs using five P-value thresholds (Pts, < 0.01, < 0.05, < 0.1, < 0.5, < 1) from GWAS reference sets. **a)** Correlation between PRS for autism spectrum disorder (ASD) and Social Responsiveness Scale at pre-treatment (SRS-pre). The blue line represents the linear model fitted by PRS for ASD and SRS score at pre-treatment. The grey curve shows 95% confidence interval of predicted linear model. **b)** The distribution of PRS for ASD in individuals with and without clinically significant copy number variations (CNVs). The center line in each box represents median value of PRS for ASD. The lower and upper box limits are the first and third quantiles. Each whisker means 1.5x interquartile range. The black points indicate outliers. **c)** The distribution of PRS for attention-deficit hyperactivity disorder (ADHD) in individuals with and without ADHD. The center line in each box represents median value of PRS for ADHD. The lower and upper box limits are the first and third quantiles. Each whisker means 1.5x interquartile range. The black points indicate outliers. **d)** Correlation between PRS for educational attainment (EA) and IQ level. The blue line represents the linear model fitted by PRS for EA and IQ level. The grey curve shows 95% confidence interval of predicted linear model.

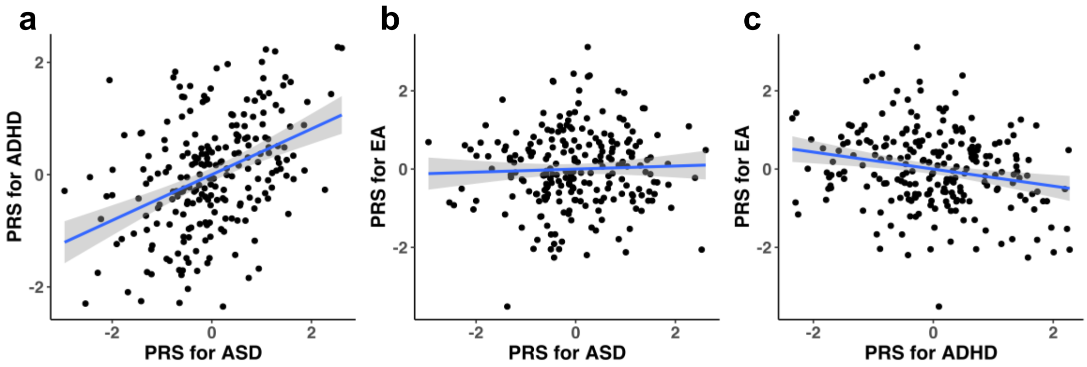

Supplementary Figure 2. Correlation between polygenic risk scores (PRSs) of autism spectrum disorder (ASD), attention deficit hyperactivity disorder (ADHD), and education attainment (EA). **a)** correlation between PRS for ASD (P-value threshold (Pt) $_{0.5}$) and PRS for ADHD (Pt $_{1.0}$). **b)** correlation between PRS for ASD (Pt $_{0.5}$) and PRS for EA (Pt $_{1.0}$). **c)** correlation between PRS for ADHD (Pt $_{1.0}$) and PRS for EA (Pt $_{1.0}$). Each blue line represents the linear model fitted by y-axis variable and x-axis variable. Each grey curve shows 95% confidence interval of predicted linear model.

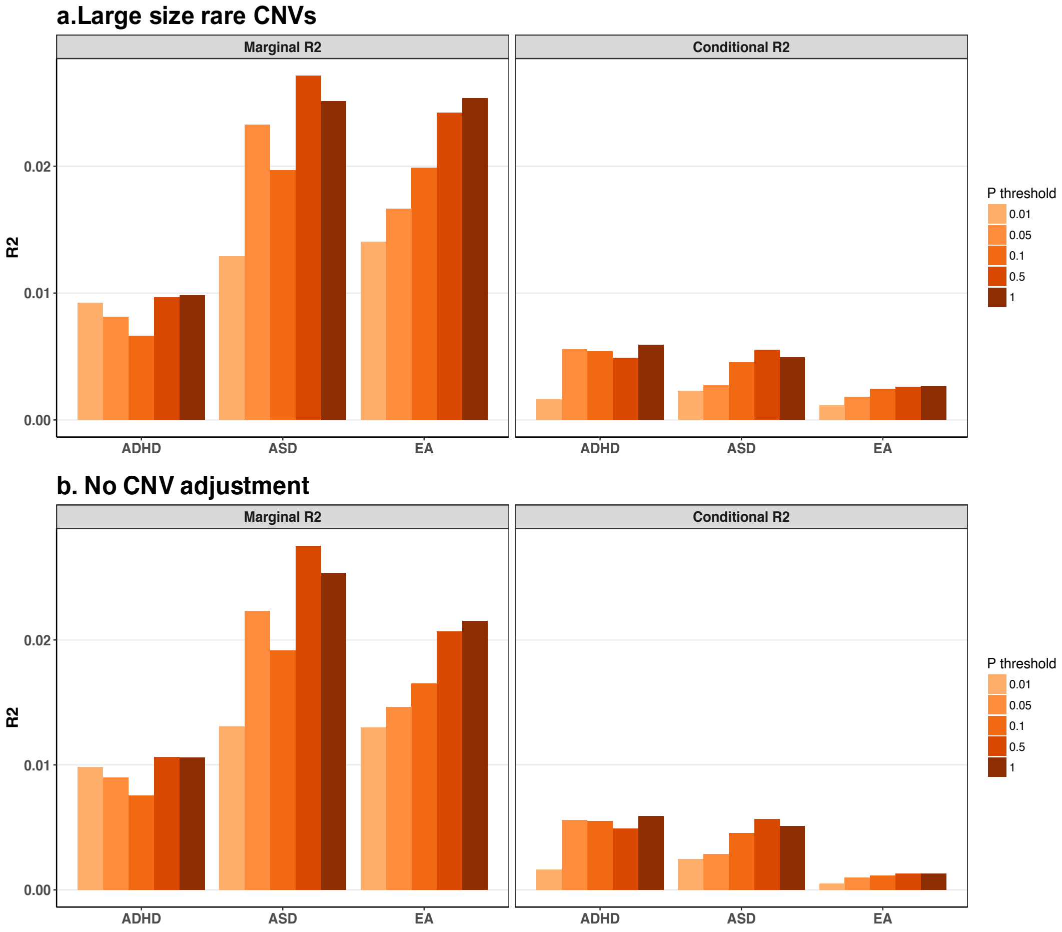

Supplementary Figure 3. Proportion of variance explained (R^2) by polygenic risk score (PRS) of autism spectrum disorder (ASD), attention deficit hyperactivity disorder (ADHD), and education attainment (EA) in intervention outcomes derived using five P-value thresholds (Pts, < 0.01, < 0.05, < 0.1, < 0.5, < 1). Marginal R^2 and conditional R^2 were calculated representing the variance explained by only fixed effects as well as the sum of fixed and random effects. **a)** R^2 by PRS in the model adjusting for the carrier status of large size (> 500kb) rare copy number variations (CNVs). **b)** R^2 by PRS in the model without CNV adjustment.

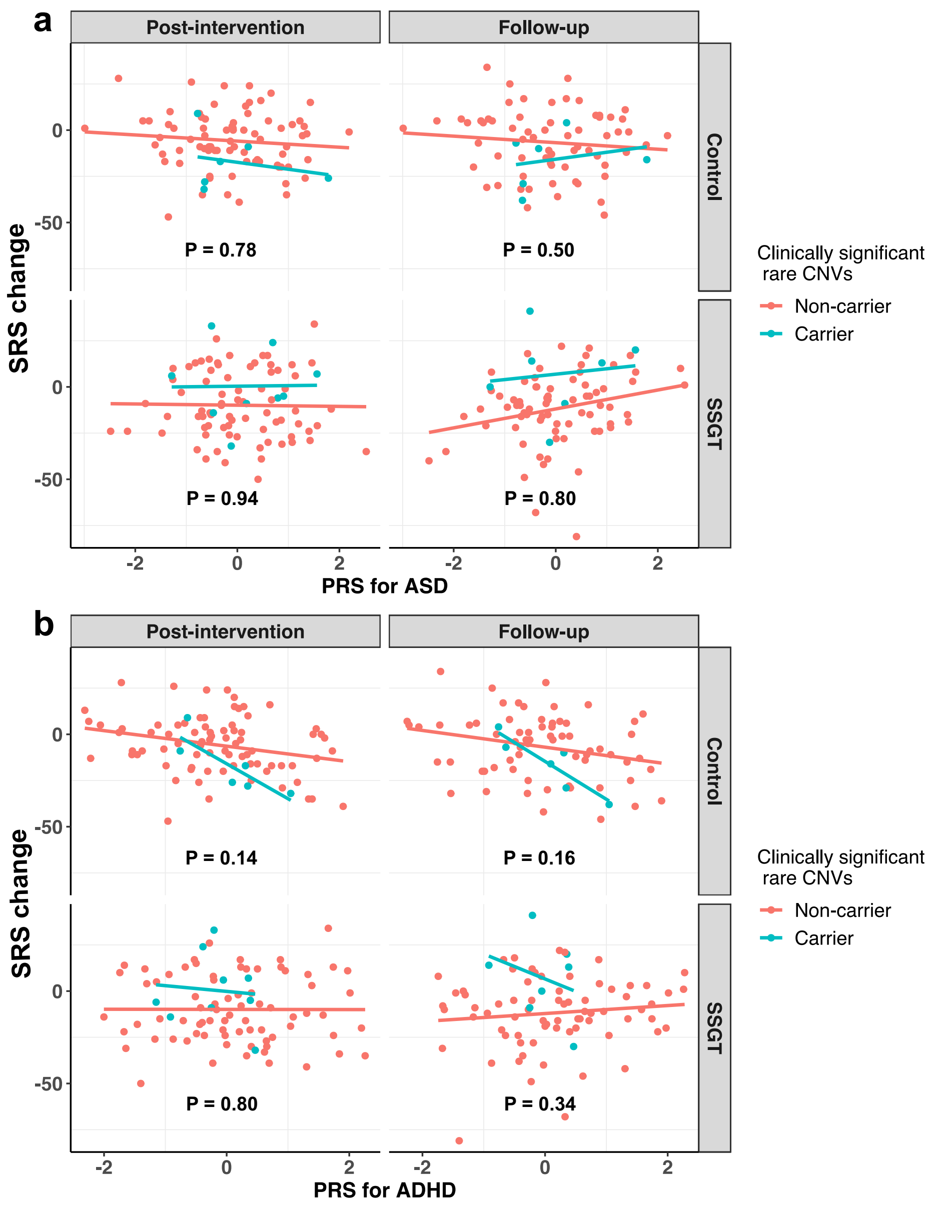

Supplementary Figure 4. The relation between Social Responsiveness Scale (SRS) changes of control and social skills group training (SSGT) groups and polygenic risk scores (PRSs) for autism spectrum disorder (ASD) and attention deficit hyperactivity disorder (ADHD) in carriers and non-carriers of clinically significant copy number variations (CNVs) at post-intervention and follow-up. **a)** changes of SRS and PRS for ASD (P-value threshold (Pt) $_{0.5}$) separated by clinically significant CNVs. **b)** changes of SRS and PRS for ADHD (Pt $_{1.0}$) separated by clinically significant CNVs. Each line represents the linear model fitted by SRS change and PRS for ASD or PRS for ADHD.

Supplementary Tables

Supplementary Table 1. Comparison of sample characteristics between total sample and genotyping sample

	Total clinical sample		Genotyping sample		P
	SSGT	Standard care	SSGT	Standard care	
Sex					
Female (n (%))	43 (14.53)	45 (15.20)	26 (13.83)	26 (13.83)	1.000
Male (n (%))	107 (36.15)	101 (34.12)	73 (38.83)	63 (33.51)	0.768
Age					
Adolescents (n (%))	67 (22.64)	57 (19.26)	56 (29.79)	60 (31.91)	0.446
Children (n (%))	83 (28.04)	89 (30.07)	43 (22.87)	29 (15.43)	0.135
IQ (Mean (sd))	96.95 (13.45)	98.13 (12.91)	98.6 (13.31)	98.26 (13.54)	0.466

Abbreviations:
SSGT: social skills group training; sd: standard deviation. n: the number of samples in each category.

Supplementary Table 2. Association between polygenic risk score (PRS) and social responsive scale (SRS) using mixed linear model adjusted for clinically significant rare copy number variations (CNVs), large size (> 500kb) rare CNVs and without any CNV adjustment.

	Clinically significant rare CNVs carrier status				Large size rare CNVs carrier status				No CNV adjustment			
	Beta	lower CI	upper CI	P	Beta	lower CI	upper CI	P	Beta	lower CI	upper CI	P
ASD PRS												
0.010												
*Post	-1.705	-5.383	1.973	0.3642	-1.692	-5.369	1.986	0.3680	-1.687	-5.364	1.991	0.3694
*Follow-up	2.194	-1.667	6.054	0.2662	2.202	-1.658	6.062	0.2644	2.214	-1.646	6.074	0.2618
*SSGT	4.274	-2.945	11.492	0. 2471	3.918	-3.361	11.196	0.2926	3.857	-3.440	11.153	0.3013
*Post*SSGT	1.139	-4.150	6.428	0.6733	1.136	-4.153	6.426	0.6740	1.098	-4.192	6.387	0.6845
*Follow-up*SSGT	-0.758	-6.197	4.681	0.7849	-0.771	-6.211	4.668	0.7812	-0.825	-6.265	4.615	0.7664
0.050												
*Post	-2.229	-6.117	1.660	0.2621	-2.223	-6.110	1.665	0.2634	-2.212	-6.100	1.677	0.2658
*Follow-up	1.098	-2.932	5.127	0.5938	1.091	-2.938	5.121	0.5959	1.113	-2.917	5.143	0.5886
*SSGT	5.516	-1.730	12.761	0.1370	5.836	-1.462	13.133	0.1184	5.705	-1.624	13.034	0.1284
*Post*SSGT	1.198	-4.153	6.549	0.6611	1.240	-4.112	6.591	0.6502	1.193	-4.159	6.545	0.6624
*Follow-up*SSGT	0.895	-4.608	6.398	0.7501	0.926	-4.577	6.430	0.7417	0.867	-4.637	6.370	0.7578
0.100												
*Post	-2.681	-6.469	1.106	0.1663	-2.679	-6.466	1.108	0.1666	-2.667	-6.455	1.120	0.1685
*Follow-up	-0.482	-4.305	3.341	0.8050	-0.489	-4.312	3.334	0.8021	-0.474	-4.297	3.350	0.8084
*SSGT	5.266	-1.972	12.505	0.1552	5.590	-1.693	12.874	0.1339	5.522	-1.787	12.832	0.1400
*Post*SSGT	1.975	-3.273	7.222	0.4614	2.017	-3.230	7.265	0.4517	1.975	-3.273	7.223	0.4612
*Follow-up*SSGT	3.672	-1.690	9.034	0.1805	3.695	-1.667	9.058	0.1778	3.647	-1.716	9.010	0.1835
0.500												
*Post	-1.990	-5.702	1.722	0.2941	-1.986	-5.698	1.726	0.2951	-1.975	-5.686	1.737	0.2979

*Follow-up	-1.427	-5.179	2.325	0.4565	-1.429	-5.181	2.322	0.4558	-1.413	-5.165	2.339	0.4609
*SSGT	5.623	-1.603	12.849	0.1286	5.767	-1.513	13.047	0.1219	5.770	-1.533	13.073	0.1228
*Post*SSGT	2.341	-2.970	7.651	0.3883	2.385	-2.926	7.695	0.3795	2.336	-2.974	7.647	0.3892
*Follow-up*SSGT	6.467	1.106	11.828	0.0187*	6.500	1.138	11.862	0.0181*	6.439	1.077	11.801	0.0192*
1.000												
*Post	-1.842	-5.587	1.904	0.3360	-1.838	-5.583	1.907	0.3368	-1.825	-5.570	1.920	0.3402
*Follow-up	-1.335	-5.123	2.453	0.4904	-1.338	-5.126	2.449	0.4891	-1.321	-5.109	2.467	0.4947
*SSGT	5.671	-1.581	12.922	0.1267	5.837	-1.468	13.142	0.1187	5.791	-1.540	13.121	0.1229
*Post*SSGT	1.724	-3.603	7.051	0.5263	1.767	-3.560	7.095	0.5161	1.714	-3.613	7.042	0.5287
*Follow-up*SSGT	5.935	0.556	11.313	0.0313*	5.973	0.595	11.352	0.0302*	5.909	0.531	11.288	0.0320*
ADHD PRS												
0.010												
*Post	-2.933	-6.870	1.003	0.1452	-2.930	-6.866	1.006	0.1456	-2.921	-6.857	1.016	0.1469
*Follow-up	-3.366	-7.639	0.907	0.1236	-3.361	-7.634	0.912	0.1241	-3.338	-7.611	0.936	0.1268
*SSGT	-3.985	-11.266	3.296	0.2845	-4.042	-11.389	3.305	0.2821	-4.342	-11.713	3.030	0.2495
*Post*SSGT	4.064	-1.146	9.274	0.1273	4.061	-1.149	9.272	0.1275	4.046	-1.165	9.256	0.1291
*Follow-up*SSGT	4.647	-0.877	10.172	0.1002	4.642	-0.883	10.167	0.1006	4.600	-0.925	10.126	0.1037
0.050												
*Post	-5.251	-9.143-	1.359	0.0086*	-5.246	-9.137	-1.354	0.0087*	-5.238	-9.130	-1.345	0.0088*
*Follow-up	-5.720	-9.818	-1.622	0.0066*	-5.717	-9.814	-1.619	0.0066*	-5.703	-9.801	-1.605	0.0067*
*SSGT	-3.787	-11.214	3.639	0.3186	-3.830	-11.323	3.664	0.3176	-4.054	-11.561	3.453	0.2910
*Post*SSGT	6.222	1.039	11.406	0.0192*	6.229	1.046	11.413	0.0191*	6.215	1.031	11.399	0.0194*
*Follow-up*SSGT	6.935	1.537	12.332	0.0123*	6.950	1.552	12.347	0.0121*	6.925	1.527	12.323	0.0124*
0.100												
*Post	-5.178	-8.936	-1.419	0.0073*	-5.173	-8.931	-1.415	0.0074*	5.166	-8.924	-1.407	0.0074*
*Follow-up	-5.448	-9.424	-1.471	0.0076*	-5.451	-9.427	-1.474	0.0076*	-5.440	-9.417	-1.463	0.0077*

*SSGT	-3.880	-11.221	3.461	0.3013	-4.016	-11.425	3.393	0.2892	-4.293	-11.723	3.138	0.2587
*Post*SSGT	6.223	1.130	11.316	0.0172*	6.223	1.131	11.316	0.0172*	6.210	1.117	11.303	0.0174*
*Follow-up*SSGT	5.748	0.409	11.087	0.0356*	5.771	0.432	11.110	0.0349*	5.751	0.412	11.091	0.0355*
0.500												
*Post	-4.521	-8.282	-0.760	0.0191*	-4.515	-8.275	-0.754	0.0192*	-4.507	-8.268	-0.746	0.0194*
*Follow-up	-4.909	-8.873	-0.945	0.0158*	-4.908	-8.872	-0.944	0.0158*	-4.899	-8.863	-0.934	0.0160*
*SSGT	-3.327	-10.725	4.071	0.3791	-3.948	-11.404	3.508	0.3005	-4.227	-11.708	3.254	0.2692
*Post*SSGT	4.539	-0.637	9.714	0.0866	4.530	-0.646	9.705	0.0872	4.503	-0.673	9.679	0.0891
*Follow-up*SSGT	6.003	0.592	11.413	0.0304*	6.030	0.619	11.440	0.0297*	5.995	0.584	11.406	0.0306*
1.000												
*Post	-4.747	-8.467	-1.027	0.0129*	-4.741	-8.461	-1.021	0.0130*	-4.734	-8.455	-1.014	0.0131*
*Follow-up	-5.309	-9.224	-1.395	0.0083*	-5.309	-9.224	-1.395	0.0082*	-5.301	-9.216	-1.386	0.0084*
*SSGT	-3.069	-10.439	4.301	0.4152	-3.633	-11.066	3.799	0.3390	-3.886	-11.343	3.570	0.3081
*Post*SSGT	4.699	-0.453	9.852	0.0748	4.696	-0.456	9.848	0.0750	4.667	-0.485	9.820	0.0768
*Follow-up*SSGT	6.669	1.296	12.042	0.0155*	6.704	1.330	12.077	0.0150*	6.665	1.291	12.039	0.0156*
ADHD PRS (with ADHD comorbidity in the model)												
0.010												
*Post	-2.933	-6.870	1.004	0.1452	-2.930	-6.866	1.007	0.1456	-2.920	-6.857	1.017	0.1470
*Follow-up	-3.365	-7.639	0.908	0.1237	-3.360	-7.633	0.914	0.1243	-3.336	-7.610	0.938	0.1270
*SSGT	-3.993	-11.298	3.312	0.2852	-4.046	-11.418	3.326	0.2832	-4.343	-11.739	3.054	0.2510
*Post*SSGT	4.059	-1.148	9.274	0.1278	4.059	-1.152	9.269	0.1278	4.042	-1.169	9.253	0.1295
*Follow-up*SSGT	4.640	-0.878	10.172	0.1008	4.640	-0.886	10.165	0.1008	4.597	-0.929	10.123	0.1039
0.050												
*Post	-5.251	-9.143	-1.358	0.0086*	-5.245	-9.137	-1.353	0.0087*	-5.237	-9.130	-1.345	0.0088*
*Follow-up	-5.718	-9.816	-1.620	0.0066*	-5.715	-9.812	-1.617	0.0066*	-5.701	-9.799	-1.603	0.0068*
*SSGT	-3.765	-11.268	3.739	0.3265	-3.782	-11.353	3.789	0.3286	-4.000	-11.585	3.585	0.3024

*Post*SSGT	6.226	1.036	11.404	0.0192*	6.226	1.042	11.409	0.0192*	6.212	1.027	11.396	0.0195*
*Follow-up*SSGT	6.946	1.534	12.330	0.0122*	6.946	1.548	12.344	0.0122*	6.921	1.523	12.320	0.0125*
0.100												
*Post	-5.177	-8.936	-1.419	0.0073*	-5.173	-8.931	-1.415	0.0074*	-5.165	-8.931	-1.415	0.0074*
*Follow-up	-5.447	-9.423	-1.470	0.0076*	-5.449	-9.426	-1.473	0.0076*	-5.439	-9.426	-1.473	0.0077*
*SSGT	-3.868	-11.293	3.557	0.3084	-3.969	-11.464	3.525	0.3004	-4.242	-11.464	3.525	0.2698
*Post*SSGT	6.221	1.129	11.315	0.0172*	6.221	1.128	11.314	0.0172*	6.208	1.115	11.301	0.0175*
*Follow-up*SSGT	5.768	0.407	11.086	0.0350*	5.768	0.429	11.107	0.0350*	5.749	0.409	11.089	0.0356*
0.500												
*Post	-4.521	-8.282	-0.760	0.0191*	-4.515	-8.275	-0.754	0.0192*	-4.507	-8.275	-0.754	0.0194*
*Follow-up	-4.909	-8.874	-0.945	0.0158*	-4.908	-8.872	-0.944	0.0158*	-4.899	-8.872	-0.944	0.0160*
*SSGT	-3.372	-10.851	4.107	0.3778	-3.964	-11.504	3.576	0.3039	-4.235	-11.504	3.576	0.2736
*Post*SSGT	4.529	-0.637	9.715	0.0873	4.529	-0.647	9.705	0.0873	4.503	-0.674	9.679	0.0892
*Follow-up*SSGT	6.029	0.593	11.414	0.0297*	6.029	0.618	11.440	0.0297*	5.994	0.583	11.406	0.0307*
1.000												
*Post	-4.747	-8.467	-1.027	0.0129*	-4.741	-8.461	-1.021	0.0130*	-4.735	-8.461	-1.021	0.0131*
*Follow-up	-5.309	-9.224	-1.394	0.0083*	-5.309	-9.224	-1.394	0.0083*	-5.300	-9.224	-1.394	0.0084*
*SSGT	-3.103	-10.555	4.348	0.4153	-3.638	-11.154	3.879	0.3439	-3.882	-11.154	3.879	0.3140
*Post*SSGT	4.695	-0.453	9.852	0.0751	4.695	-0.457	9.847	0.0751	4.666	-0.487	9.819	0.0769
*Follow-up*SSGT	6.703	1.295	12.043	0.0150*	6.703	1.329	12.076	0.0150*	6.664	1.289	12.038	0.0156*

Abbreviations:
ASD: Autism spectrum disorder; ADHD: Attention deficit hyperactivity disorder; SSGT: social skills group training; CI: confidence interval
*: P < 0.05.

Supplementary Table 3. Association between polygenic risk score (PRS) and intervention outcomes using mixed linear model adjusted for clinically significant rare copy number variations (CNVs) in social skills group training (SSGT) and standard care groups

	Beta	lower CI	upper CI	P
SSGT group				
ASD PRS 0.50				
*Post	0.430	-3.613	4.473	0.835
*Follow-up	5.145	1.068	9.221	0.0144*
ADHD PRS 1.00				
*Post	0.0239	-3.854	3.902	0.990
*Follow-up	1.364	-2.649	5.367	0.505
Standard care group				
ASD PRS 0.50				
*Post	-1.953	-5.403	1.497	0.269
*Follow-up	-1.346	-4.834	2.141	0.450
ADHD PRS 1.00				
*Post	-4.729	-8.086	-1.372	0.00647*
*Follow-up	-5.277	-8.810	-1.744	0.00394*

Abbreviations:
CI: confidence interval.
*: P < 0.05

Supplementary Table 4. Analyses of 31 gene sets on intervention outcomes at post-intervention and follow-up. The models are adjusted for clinically significant rare copy number variations (CNVs) and large size (> 500kb) rare CNVs

Gene sets	Number of genes	Model adjusted for clinically significant rare CNVs carrier status								Model adjusted for large size rare CNVs carrier status							
		post-intervention				Follow-up				post-intervention				Follow-up			
		Beta	Se	P	Corrected P	Beta	Se	P	Corrected P	Beta	Se	P	Corrected P	Beta	Se	P	Corrected P
Gene set group: Synaptic																	
Cell adhesion and trans-synaptic signaling	74	0.25	0.10	0.009*	0.234	-0.09	0.10	0.812	1.000	0.26	0.10	0.007*	0.199	-0.06	0.10	0.720	1.000
Cell metabolism	49	-0.02	0.11	0.558	1.000	-0.03	0.11	0.613	1.000	0.05	0.11	0.341	1.000	0.00	0.11	0.497	1.000
Endocytosis	26	-0.20	0.18	0.873	1.000	0.03	0.18	0.437	1.000	-0.20	0.18	0.873	1.000	0.03	0.18	0.442	1.000
Excitability	55	0.25	0.13	0.026*	0.538	0.15	0.13	0.125	0.978	0.27	0.13	0.017*	0.411	0.14	0.13	0.127	0.978
Exocytosis	76	0.11	0.10	0.124	0.976	0.00	0.10	0.501	1.000	0.10	0.10	0.160	0.993	-0.02	0.10	0.579	1.000
GPCR signaling	40	0.25	0.13	0.024	0.514	-0.03	0.13	0.593	1.000	0.26	0.13	0.023	0.501	-0.03	0.13	0.600	1.000
G-protein Relay	26	0.17	0.18	0.166	0.994	-0.12	0.19	0.735	1.000	0.08	0.18	0.326	1.000	-0.13	0.19	0.752	1.000
Intracellular signal transduction	138	0.20	0.07	0.003*	0.084	-0.03	0.07	0.660	1.000	0.20	0.07	0.003*	0.083	-0.05	0.07	0.737	1.000
Intracellular trafficking	67	-0.24	0.10	0.993	1.000	-0.02	0.09	0.591	1.000	-0.25	0.10	0.995	1.000	0.00	0.09	0.510	1.000
Ion balance/transport	39	-0.13	0.13	0.841	1.000	-0.08	0.13	0.716	1.000	-0.12	0.13	0.815	1.000	-0.06	0.13	0.672	1.000
Ligand-gated ion channel signaling	32	-0.16	0.17	0.831	1.000	-0.09	0.17	0.708	1.000	-0.16	0.17	0.829	1.000	-0.10	0.17	0.723	1.000
Neurotransmitter metabolism	25	-0.24	0.19	0.897	1.000	0.11	0.19	0.280	1.000	-0.23	0.19	0.893	1.000	0.11	0.19	0.277	1.000
Peptide/neurotrophin signals	25	-0.20	0.18	0.870	1.000	-0.12	0.18	0.747	1.000	-0.20	0.18	0.871	1.000	-0.11	0.17	0.741	1.000
Protein cluster	41	0.05	0.14	0.357	1.000	-0.04	0.13	0.612	1.000	0.01	0.14	0.465	1.000	-0.05	0.13	0.643	1.000
RPSFB	62	0.06	0.10	0.263	1.000	0.19	0.10	0.030*	0.587	0.05	0.10	0.289	1.000	0.18	0.10	0.030*	0.593
Structural plasticity	87	0.01	0.09	0.434	1.000	-0.08	0.09	0.830	1.000	0.01	0.09	0.450	1.000	-0.09	0.09	0.856	1.000

Tyrosine kinase signaling	7	-0.21	0.35	0.721	1.000	0.15	0.35	0.328	1.000	-0.08	0.35	0.595	1.000	0.20	0.35	0.280	1.000
Unknown	53	0.16	0.12	0.092	0.936	0.13	0.11	0.121	0.975	0.18	0.12	0.066	0.860	0.15	0.11	0.079	0.907
Synaptic:Sanders FDR0.01 large synaptic	18	-0.26	0.19	0.921	1.000	-0.23	0.20	0.877	1.000	-0.27	0.19	0.922	1.000	-0.24	0.20	0.882	1.000
Gene set group: Glia																	
Glia astrocytes Duncan	34	-0.13	0.14	0.816	1.000	-0.05	0.14	0.630	1.000	-0.05	0.14	0.636	1.000	-0.04	0.14	0.602	1.000
Glia oligocytes Duncan	42	0.12	0.13	0.175	0.995	-0.04	0.13	0.612	1.000	0.13	0.13	0.145	0.987	-0.01	0.13	0.535	1.000
Glia Duncan	118	0.00	0.08	0.504	1.000	-0.04	0.08	0.711	1.000	0.02	0.08	0.423	1.000	-0.05	0.08	0.722	1.000
Gene set group: FMRP targets																	
FMRP_targets Ascano_937	869	0.01	0.03	0.400	1.000	0.01	0.03	0.431	1.000	0.00	0.03	0.433	1.000	0.01	0.03	0.381	1.000
FMRP_targets Darnell_782	103	-0.03	0.08	0.623	1.000	-0.04	0.08	0.689	1.000	-0.04	0.08	0.668	1.000	-0.06	0.08	0.754	1.000
Gene set group: Glutamate																	
Glutamate Duncan	140	0.08	0.07	0.140	0.987	0.00	0.07	0.498	1.000	0.08	0.07	0.136	0.983	-0.01	0.07	0.539	1.000
Gene set group: Mitochondria																	
Mitochondria Duncan	66	-0.06	0.10	0.710	1.000	-0.11	0.11	0.853	1.000	0.01	0.10	0.459	1.000	-0.09	0.11	0.815	1.000
Mitochondria crista Duncan	5	0.34	0.41	0.207	0.998	0.29	0.41	0.239	1.000	0.29	0.41	0.241	1.000	0.23	0.41	0.285	1.000
Mitochondria distribution Duncan	6	-0.72	0.27	0.996	1.000	0.16	0.29	0.283	1.000	-0.49	0.27	0.966	1.000	0.16	0.29	0.283	1.000
Mitochondria fission Duncan	11	-0.06	0.26	0.594	1.000	0.03	0.26	0.457	1.000	0.01	0.26	0.490	1.000	0.05	0.26	0.427	1.000
Mitochondria fission_plus Duncan	23	0.09	0.18	0.300	1.000	0.08	0.18	0.325	1.000	0.13	0.18	0.234	1.000	0.08	0.18	0.323	1.000
Mitochondria fusion Duncan	9	0.11	0.33	0.372	1.000	-0.22	0.32	0.755	1.000	0.09	0.33	0.395	1.000	-0.28	0.32	0.806	1.000

Abbreviations:

SSGT: social skills group training; GPCR: G-protein-coupled receptor; RPSFB: RNA and protein synthesis, folding and breakdown; Unknown: genes that are known to be expressed in the synapse but currently have no known shared function with other genes.
*: $P < 0.05$

Supplementary Table 5. Power estimation for different sample sizes of polygenic risk scores (PRSs) for autism spectrum disorder (ASD) (P-value threshold (Pt) $_{0.5}$), attention deficit hyperactivity disorder (ADHD) (Pt $_{1.0}$), and educational attainment (EA) (Pt $_{1.0}$) effect on follow-up intervention outcomes at significance level $P < 0.05$.

	PRS for ASD	PRS for ADHD	PRS for EA
**Sample sizes**			
188 (Our study sample size)	0.726	0.766	0.119
300	0.894	0.909	0.130
400	0.963	0.969	0.140
