# Supplementary material for: The influence of common polygenic risk and gene sets on social skills group training response in autism spectrum disorder
Source: NPJ Genom Med. 2020 Oct 12;5:45. doi: 10.1038/s41525-020-00152-x (PMC7550579; doi:10.1038/s41525-020-00152-x)
Supplement: Supplementary file 2 — Reporting Summary [file 41525_2020_152_MOESM2_ESM.pdf]

nature research

Corresponding author(s): Tammimies

Last updated by author(s): Jul 25, 2020

Reporting Summary

Nature Research wishes to improve the reproducibility of the work that we publish. This form provides structure for consistency and transparency in reporting. For further information on Nature Research policies, see our Editorial Policies and the Editorial Policy Checklist.

Statistics

For all statistical analyses, confirm that the following items are present in the figure legend, table legend, main text, or Methods section.

n/a	Confirmed	
☐	☒	The exact sample size (*n*) for each experimental group/condition, given as a discrete number and unit of measurement
☐	☒	A statement on whether measurements were taken from distinct samples or whether the same sample was measured repeatedly
☐	☒	The statistical test(s) used AND whether they are one- or two-sided<br>*Only common tests should be described solely by name; describe more complex techniques in the Methods section.*
☐	☒	A description of all covariates tested
☐	☒	A description of any assumptions or corrections, such as tests of normality and adjustment for multiple comparisons
☐	☒	A full description of the statistical parameters including central tendency (e.g. means) or other basic estimates (e.g. regression coefficient) AND variation (e.g. standard deviation) or associated estimates of uncertainty (e.g. confidence intervals)
☐	☒	For null hypothesis testing, the test statistic (e.g. *F*, *t*, *r*) with confidence intervals, effect sizes, degrees of freedom and *P* value noted<br>*Give P values as exact values whenever suitable.*
☒	☐	For Bayesian analysis, information on the choice of priors and Markov chain Monte Carlo settings
☒	☐	For hierarchical and complex designs, identification of the appropriate level for tests and full reporting of outcomes
☐	☒	Estimates of effect sizes (e.g. Cohen's *d*, Pearson's *r*), indicating how they were calculated

Our web collection on statistics for biologists contains articles on many of the points above.

Software and code

Policy information about availability of computer code

Data collection	We have used available software with standard codes.
Data analysis	Data from genotyping were transformed from Affymetrix .CEL format to .tped format using “Affy2sv” package v1.0.14 in R software. Quality control of the genotyping data was performed by PLINK v1.90. Ancestry of the participants was estimated using EIGENSOFT v7.2.1. haplotypes were inferred based on reference panel using SHAPEIT v2. Imputation was performed using IMPUTE2 v2.3.2. SNPs were intersected together after post-imputation QC using both SNPTEST v2.5.5 and PLINK v1.90. Polygenic risk score was calculated using PRSice v2.1.4. All statistical analysis was conducted using R v3.4.2

For manuscripts utilizing custom algorithms or software that are central to the research but not yet described in published literature, software must be made available to editors and reviewers. We strongly encourage code deposition in a community repository (e.g. GitHub). See the Nature Research guidelines for submitting code & software for further information.

Data

Policy information about availability of data

All manuscripts must include a data availability statement. This statement should provide the following information, where applicable:

- Accession codes, unique identifiers, or web links for publicly available datasets
- A list of figures that have associated raw data
- A description of any restrictions on data availability

The raw array data or phenotypic data has not been shared in a public database due to the limited ethical approval, and informed consent from the participants for data sharing are available from the corresponding author (kristiina.tammimies@ki.se) upon request and subject to necessary clearances.

Field-specific reporting

Please select the one below that is the best fit for your research. If you are not sure, read the appropriate sections before making your selection.

☒ Life sciences ☐ Behavioural & social sciences ☐ Ecological, evolutionary & environmental sciences

For a reference copy of the document with all sections, see nature.com/documents/nr-reporting-summary-flat.pdf

Life sciences study design

All studies must disclose on these points even when the disclosure is negative.

Sample size	In our study, clinical data and samples from 207 participants were selected for genotyping, and 188 of them passed genotyping quality control. Bootstrapping was used to evaluate the power of Polygenic risk score (PRS) association on intervention outcome. The power of PRSs for ADHD, ASD, and educational attainment based on our study sample size was 0.766, 0.726, and 0.119, respectively, at a significant level P < 0.05
Data exclusions	There were 188 participants who passed quality control from total 207 participants. Individuals with discordant sex, heterozygosity rate > 3SD, individual genotype failure rate > 0.03, and relatedness were removed. Ancestry of the participants was estimated using principal component analysis (PCA) based on the HapMap Phase III (HapMap3) data. We restricted our analyses to participants with European ancestry. In addition, the qualified data were combined to clean low-quality markers with the following criteria: minor allele frequency < 0.05, Hardy-Weinberg equilibrium < 1e-06, individual missingness < 0.1, and marker missingness < 0.05. The final genotype data consist of 539 106 SNPs after genotyping marker quality control
Replication	As no additional clinical and genotyping data were available for social skills group training treatment cohort, no replication was performed.
Randomization	The original data of participants were acquired from a randomized controlled trial (Choque-Olsson et al. Social Skills Training for Children and Adolescents With Autism Spectrum Disorder: A Randomized Controlled Trial. 2015.). More detailed description of trial design and randomization can be seen in this reference.
Blinding	The original data of participants were acquired from a randomized controlled trial (Choque-Olsson et al. Social Skills Training for Children and Adolescents With Autism Spectrum Disorder: A Randomized Controlled Trial. 2015.). More detailed description of blinding can be seen in this reference.

Reporting for specific materials, systems and methods

We require information from authors about some types of materials, experimental systems and methods used in many studies. Here, indicate whether each material, system or method listed is relevant to your study. If you are not sure if a list item applies to your research, read the appropriate section before selecting a response.

Materials & experimental systems

n/a	Involved in the study	
☒	☐	Antibodies
☒	☐	Eukaryotic cell lines
☒	☐	Palaeontology and archaeology
☒	☐	Animals and other organisms
☐	☒	Human research participants
☐	☒	Clinical data
☒	☐	Dual use research of concern

Methods

n/a	Involved in the study	
☒	☐	ChIP-seq
☒	☐	Flow cytometry
☒	☐	MRI-based neuroimaging

Human research participants

Policy information about studies involving human research participants

Population characteristics	Previous studies have indicated population characteristics associated with inferior social skills group training intervention outcomes were younger age (children), male sex.
Recruitment	Totally 296 children (7–12 years) and adolescents (13–17 years) with a diagnosis of autism, atypical autism, Asperger syndrome, or pervasive developmental disorder not otherwise specified using ICD-10 criteria were included in the trial. Based on the inclusion criteria for the RCT, all participants had full-scale IQ > 70 according to the Wechsler Intelligence Scale for Children and at least one common comorbid psychiatric diagnosis of ADHD, depression, or anxiety disorder according to ICD-10.
Ethics oversight	The trial and sample collection were approved by the ethical review board in Stockholm (Dnr 2012/385-31/4) and the clinical authorities of the two involved counties in our study.

Note that full information on the approval of the study protocol must also be provided in the manuscript.

Clinical data

Policy information about clinical studies

All manuscripts should comply with the ICMJE guidelines for publication of clinical research and a completed CONSORT checklist must be included with all submissions.

Clinical trial registration	The trial was registered online on ClinicalTrials.gov (NCT01854346)
Study protocol	The protocol can be found in Choque-Olsson et al. Social Skills Training for Children and Adolescents With Autism Spectrum Disorder: A Randomized Controlled Trial. 2015.
Data collection	The original multicenter, randomized pragmatic RCT of SSGT ("KONTAKT") recruited participants from 13 child and adolescent psychiatry outpatient units in Sweden between August 2012 and October 2015 (identifier: NCT01854346, registration May 2013)
Outcomes	In the study, we used the parent-reported Social Responsiveness Scale (SRS) as the primary intervention outcome measure, which is a 65-item Likert-type scale generating totals scores ranging between 0 and 195, with a higher score indicating greater autism trait severity
